# A comparison of meta-analysis results with and without adjustment for the healthy worker effect: cancer mortality among workers in the semiconductor industry

**DOI:** 10.4178/epih.e2021057

**Published:** 2021-09-08

**Authors:** Sung-Ho Hwang, Moon-Young Park, Won Jin Lee, Inho Park, Kimyong Hong, Donguk Park, Kyoung-Mu Lee

**Affiliations:** 1 National Cancer Control Institute, National Cancer Center, Goyang, Korea; 2 Department of Environmental Health, School of Public Health, Seoul University, Seoul, Korea; 3 Department of Preventive Medicine, Korea University College of Medicine, Seoul, Korea; 4 Department of Statistics, Pukyong University, Busan, Korea; 5 Department of Nursing, Songkok University, Chuncheon, Korea; 6 Department of Environmental Health, Korea National Open University, Seoul, Korea

**Keywords:** : Meta-analysis, Mortality, Semiconductor, Healthy worker effect

## Abstract

**OBJECTIVES:**

This study compared the results of meta-analysis with and without adjustment for the healthy worker effect on the association between working in the semiconductor industry and cancer mortality.

**METHODS:**

Six studies that reported standardized mortality ratios (SMRs) for cancers were selected for meta-analysis. Using a random-effects model, the SMR results from each study were combined for all cancers and leukemias to estimate the summary SMRs (95% confidence interval, CI). To adjust for the healthy worker effect, the relative standardized mortality ratio (rSMR=SMR_x_/SMR_not x_) were calculated using observed and expected counts for the specific cause of interest (i.e., all cancers and leukemias) and the observed and expected counts for all other causes of mortality. Then, the rSMR results were combined to estimate the summary rSMRs (95% CIs).

**RESULTS:**

The SMRs for all causes of mortality among semiconductor industry workers ranged from 0.25 to 0.80, which reflects a significant healthy worker effect. A remarkable difference was found between the summary SMRs and the summary rSMRs. The summary SMR for all cancers was 0.70 (95% CI, 0.63 to 0.79) whereas the summary rSMR was 1.38 (95% CI, 1.20 to 1.59). The summary SMR for leukemia was 0.88 (95% CI, 0.72 to 1.07), and the summary rSMR was 1.88 (95% CI, 1.20 to 2.95).

**CONCLUSIONS:**

Our results suggest that adjustment for the healthy worker effect (i.e., rSMR) may be useful in meta-analyses of cohort studies reporting SMRs.

## INTRODUCTION

Semiconductor manufacturing has become the core of the entire microelectronics industry. The concern of the semiconductor industry for the health of its workers includes the cancer risk from recirculated air for particle control and suspected carcinogens including ionizing radiation, asbestos, arsenic and arsenical compounds, chromium compounds, sulfuric acid mist, ultraviolet light, trichloroethylene, carbon tetrachloride, nickel, and antimony trioxide [1,2]. The semiconductor industry is known to be highly chemical-intensive, especially for the wafer fabrication process [1]. Since there were cases reported at IBM in the United States and National Semiconductor in the Unite Kingdom, a series of studies were conducted to investigate whether there exists a higher cancer risk among workers as compared to the general population. The first cohort study result was reported in 1985 by the United Kingdom Health and Safety Executive [3]. Since IBM also began an internal investigation of the cancer cluster [4], a series of epidemiological studies were launched, most of which were motivated by activism and lawsuits from workers and community residents [3,5-15]. Epidemiological evidence to date suggests that excess risk may be present for several cancers such as leukemia, brain tumor, and breast cancer for workers in the semiconductor industry. However, a definitive association between working in semiconductor manufacturing and cancers has not been found [16].

The relevance of epidemiological research reporting the standardized mortality ratios (SMRs) for workers in the semiconductor industry is often limited by the healthy worker effect. The healthy worker effect refers to the consistent tendency for actively employed people to have more favorable mortality outcomes than the population at large, and includes the effects of initial hiring into the workforce (healthy hire effect) and those of continuing employment (healthy worker survivor effect) [17]. Because healthy people tend to be working in industries of interest for research, associations are underestimated when comparing the mortality or disease incidence rates between these workers and the general population. A number of strategies have been suggested for adjusting the healthy worker effect. One of the simple correction methods is the relative standardized mortality ratio (rSMR), which is calculated as the ratio of the cause-specific SMR to the SMR for other causes, omitting the cause of interest [18,19].

In this meta-analysis of retrospective cohort studies reporting SMRs for workers in the semiconductor industry, the rSMR method was explored to adjust for the healthy worker effect and the summarized results with and without this method were compared. The purpose of this study was to compare summary SMRs and summary rSMRs in a meta-analysis of SMR studies among workers in the semiconductor industry.

## MATERIALS AND METHODS

**Figure 1.**
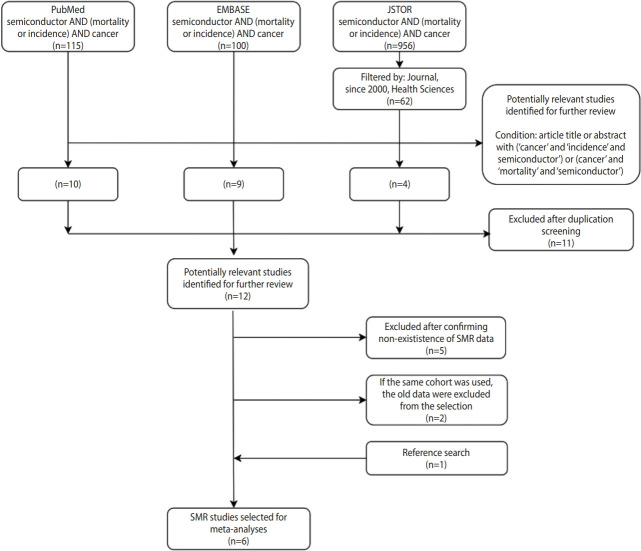
Workflow diagram for literature search and selection of standardized mortality ratio (SMR) studies for meta-analyses.

A literature search was conducted for studies on mortality for workers in the semiconductor industry using the PubMed, EMBASE, and JSTOR databases up to November 2020. Search terms were semiconductor AND (mortality OR incidence) AND cancer. The number of potentially relevant studies (i.e., cohort studies that evaluated the association between cancer mortality and working in the semiconductor industry) identified for further review was 4 from JSTOR, 9 from EMBASE, and 10 from PubMed. After excluding duplicated studies, potentially relevant studies were identified from each database (n= 11). A total of 6 studies were selected after excluding again the studies without SMR data (n= 5) and adding one study through reference search (n= 1) (Figure 1). When there were multiple reports from the same study, the most recent one was included in our analysis. Four coauthors contributed to the selection process by collaborating in the search for previous studies, but did not conduct independent reviews or compare results.

The data were combined using a random-effects model to estimate the summary SMR (95% confidence interval, CI) considering significant inter-study heterogeneity. Heterogeneity among the studies was evaluated as I^2^ and statistical significance evaluated by the Cochrane Q-test. Publication bias was assessed using the Egger test. All meta-analyses were conducted using the meta package in R version 4.0.0.

By applying methods described in the report from the Agricultural Health Study [18], the rSMR (rSMR= SMR_x_/SMR_not x_) was calculated for all cancers and leukemias by comparing the ratio between observed and expected counts for the specific cause of mortality (i.e., O_x_ and E_x_) and the ratio between observed and expected counts for the other causes of mortality (i.e., O_not x_ and E_not x_). The reference values were calculated as observed (or expected) counts for all causes minus observed (or expected) values for the specific cause. This measure is in essence the ratio of 2 Poisson variables for mutually exclusive events. When O_total_ and E_total_ is defined as O_x_+O_y_, and E_x_+E_y_, respectively, the rSMR and its CI (i.e., rSMR_LL_ and rSMR_UL_, denoting lower and upper confidence limits, respectively) are expressed as follows:


$$rSMR=\frac{SMR_{x}}{SMR_{y}}=\frac{O_{x}/E_{x}}{O_{y}/E_{y}}$$

where


$$rSMR_{LL}=\frac{\left\{\frac{O_{x}}{O_{x}+(O_{y}+1)\times F(2O_{y}+2,2O_{x};\frac{a}{2})}\times E_{y}\times \frac{\left(O_{x}+O_{y}\right)}{\left(E_{x}+E_{y}\right)}\right\}}{\left\{(1-\frac{O_{x}}{O_{x}+(O_{y}+1)\times F(2O_{y}+2,2O_{x};\frac{a}{2})})\times E_{x}\times \frac{\left(O_{x}+O_{y}\right)}{\left(E_{x}+E_{y}\right)}\right\}}$$

and


$$rSMR_{UL}=\frac{\{\frac{\left(O_{x}+1)\times F(2O_{x}+2,2O_{y};\frac{a}{2}\right)}{O_{y}+(O_{x}+1)\times F(2O_{x}+2,2O_{y};\frac{a}{2})}\}\times E_{y}\times \frac{\left(O_{x}+O_{y}\right)}{\left(E_{x}+E_{y}\right)}}{\left\{(1-\frac{\left(O_{x}+1\right)\times F\left(2O_{x}+2,2O_{y};\frac{a}{2}\right)}{O_{y}+\left(O_{x}+1\right)\times F\left(2O_{x}+2,2O_{y};\frac{a}{2}\right)})\times E_{x}\times \frac{\left(O_{x}+O_{y}\right)}{\left(E_{x}+E_{y}\right)}\right\}}$$

In the equation, F denotes an F-inverse function to obtain a point (i.e., F-value) from an F-distribution given 2 degrees of freedom and a p-value (α/2). When both the unadjusted SMR and adjusted SMR were reported, it was decided to adopt the adjusted SMR rather than the unadjusted SMR. Observed counts (i.e., deaths) and SMR values were retrieved from each study selected, and expected counts were calculated using SMRs and observed counts. For several studies, it was necessary to restrict the results to men or women.

**Figure 2.**
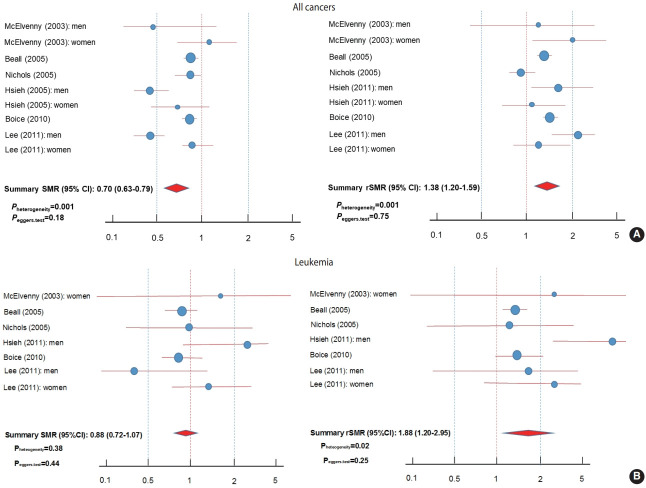
Comparison of SMRs and rSMRs for all cancers (A) and leukemia (B) in a meta-analysis of retrospective cohort studies reporting SMRs for workers in the semiconductor industry. p_heterogeneity_ was calculated by the Cochrane Q-test and p_eggers.test_ was calculated by the Egger test. SMR, standardized mortality ratio; rSMR, relative standardized mortality ratio; CI, confidence interval.

The same meta-analytic procedures were adopted to estimate the summary rSMR and 95% CI for all cancers and leukemias to compare with the corresponding summary SMR and 95% CI. Forest plots were then used to collectively show the SMRs and the rSMRs for comparison (Figure 2). Although leukemia, brain tumor, and breast cancer were listed as possibly associated with working in the semiconductor industry, leukemia was the only subtype of cancer for which a meta-analysis was conducted in our study. For brain tumor, 2 out of the 6 selected SMR studies did not provide data for brain tumors and 2 studies reported brain tumor data as tumors of the central nervous system (a broader category that includes brain tumors). For breast cancer, only 2 studies reported data for women. For reference, p_heterogeneity_ and p_eggers.test_ were also included in Figure 2.

## RESULTS

The meta-analysis of those 6 studies included 364,263 men and women workers in total with a follow-up of more than 4.6 million person-years. The SMR values for all causes ranged from 0.27 to 0.80, suggesting strong healthy worker effects for the selected studies (Table 1). However, the summarized risk tended to increase (i.e., to be shifted to the right in the forest plots) when the healthy worker effect was taken into account by using the rSMRs calculated for all cancers and leukemias (Table 2 and Figure 2).

### All cancers

The summary SMR and 95% CI for the association between working in the semiconductor industry and all cancers was 0.70 (95% CI, 0.63 to 0.79; p< 0.001). Inter-study heterogeneity was 69% (p_heterogeneity_= 0.001) and no significant publication bias was found (p_eggers.test_= 0.18).

When the rSMRs were summarized, a significant positive association was found (summary rSMR, 1.38; 95% CI, 1.20 to 1.59, p< 0.001) (Table 2 and Figure 2A). Thus, an approximate 1.9-fold increase in risk was observed for all cancers. Inter-study heterogeneity was 70% (p_heterogeneity_= 0.001) and no significant publication bias was found (p_eggers.test_= 0.75).

### Leukemia

For leukemia, the summary SMR (95% CI) for the association between working in the semiconductor industry and leukemia risk was 0.88 (95% CI, 0.72 to 1.08; p= 0.21). No significant interstudy heterogeneity or publication bias was found (I^2^: 7% and p_heterogeneity_= 0.38; p_eggers.test_= 0.44).

When the rSMRs were summarized, a significant positive association was found (summary rSMR, 1.88; 95% CI, 1.20 to 2.95; p< 0.001) (Table 2 and Figure 2B). Thus, an approximate 1.5-fold increase in risk was observed for leukemia. Inter-study heterogeneity was 61% (p_heterogeneity_= 0.02) and no significant publication bias was found (p_eggers.test_= 0.25).

## DISCUSSION

We adopted the rSMR method to adjust for the healthy worker effect in a meta-analysis of 6 retrospective cohort studies which evaluated whether semiconductor industry workers are at increased risk of cancer mortality. After adjusting for the healthy worker effect, we found that there were significant associations between working in the semiconductor industry and increased cancer mortality.

It should be noted that previous studies on mortality or incidence of cancer among semiconductor workers have mentioned the healthy worker effect as one of their limitations [6,9,11,14]. There have also been studies addressing the healthy worker effect when estimating risks of disease occurrence related to occupational asbestos exposure, the trucking industry, and autoworkers [20-22]. In the same context, a direct comparison of semiconductor cohorts with the general population may be inappropriate because the general good health of semiconductor workers may mask potential adverse health effects of the semiconductor industry. Waggoner et al. [18] adopted the rSMR analysis to adjust for the healthy worker effect in the Agricultural Health Study. Although the cohort experienced a lower mortality rate overall when compared with the general population, relatively higher rates of death from various cancers (e.g., lymphohematopoietic cancers, melanoma, and malignancies of the digestive system, prostate, kidney, brain, thyroid, eye, and ovary) among pesticide applicators was found after adjusting for the lower mortality of the cohort than the general population. Likewise, the rSMR analysis of cancer mortality in our study identified some aspects that were potentially masked in the SMR analysis.

Many different types of statistical adjustment for the healthy worker effect including the rSMR analysis had already been developed: for example, setting a minimum follow-up duration, using a lag time (at least 2-3 years) to adjust for the healthy worker effect, G-estimation methods, and others [23-26]. Among them, the rSMR method has the advantage of easily adjusting the healthy worker effect using observed and expected counts retrievable from previously published SMR papers, and also of being combined in the meta-analysis.

Given that the results of the SMR and rSMR analyses were very much in contrast, the results from the SMR analysis should not be regarded as conclusive. According to Lee et al. [14], the overall mortality of workers in the semiconductor industry in Korea was much lower than that of the general Korean population and no significant increase was observed in terms of mortality or incidence of leukemia, which was the main cancer type reported among workers in the largest semiconductor company in Korea. In contrast, the incidence of non-Hodgkin lymphoma in women workers and thyroid cancer in men workers was found to be significantly elevated. The results of this study suggest that the rSMR analysis should be considered in determining job-relatedness.

In this study, limitations of the meta-analysis include the small number of studies analyzed and the inherent weakness of an SMR study. The inter-study heterogeneity of all cancers can be attributed in part to the types of cancer included in each study and whether the reported data were classified by gender. Therefore, a random-effect model was used to estimate summary measures. The SMR studies did not control for potential confounding factors and the follow-up period tended to be short. Moreover, the rSMR method cannot use the standardized incidence ratio (SIR), which may be a better measure of risk than SMR; therefore, studies reporting only the SIR were excluded from our study.

Despite these limitations, our results show that the rSMR method may be useful in determining summary risk adjusted for the healthy worker effect through meta-analysis.

In conclusion, our results suggest that the healthy worker effect may be partly adjusted by summarizing the rSMRs in a meta-analysis of cohort studies reporting SMRs. Other types of statistical adjustment could be evaluated for application in future epidemiological study designs.

## Figures and Tables

**Table 1.** Retrospective cohort studies that evaluated mortality among workers involved in semiconductor industry

Study	Country	Cohort and follow-up	Age	Subgroup	Death of all causes		All cancers		Leukemia	
					Observed	SMR (95% CI)	Observed	SMR (95% CI)	Observed	SMR (95% CI)
McElvenny et al., 2003 [6]^2^	UK	Semiconductor workers at Edinburgh, Scotland (n=4,388; 1970-1990)	49% workers aged <25 yr	Men workers (n=2,126)	27	0.40 (0.27, 0.59)^1^	6	0.47 (0.17, 1.02)^1^	-	-
		End of follow-up: Dec. 31, 2000	84% workers aged <35 yr	Women workers (n=2,262)	44	0.75 (0.54, 1.01)^1^	23	1.10 (0.69, 1.64)^1^	1	1.72 (0.04, 9.61)^1^
		Mean length of follow-up: 12.5 yr								
Beall et al., 2005 [7]	USA	Workers at semiconductor facilities at East Fishkill, Burlington, and San Jose (IBM) (n=126,836, 1965-1999)	Median age was 44 yr		6,579	0.65 (0.64, 0.67)	2,159	0.78 (0.75, 0.81)	91	0.85 (0.69, 1.05)
		Follow-up: 2,055,328 PYs								
Nichols et al., 2005 [9]^3^	UK	Employees from a semiconductor factory (West Midlands) (n=1807; 1970-2002)			307	0.80 (0.72, 0.89)	111	0.77 (0.63, 0.92)	3	0.96 (0.20, 2.82)
Hsieh et al., 2005 [8]	Taiwan	Workers in eight semiconductor companies in Taiwan (men: 19,816, women: 27,610; 1980-2000)		Men workers (n=18,816)	98	0.27 (0.22, 0.33)	27	0.41 (0.27, 0.60)	7	2.18 (0.87, 4.49)
				Women workers (n=27,610)	93	0.63 (0.51, 0.77)	23	0.68 (0.42, 1.02)	-	-
Boice et al., 2010 [11]	US	Workers employed in semiconductor industry (1968-2002, n=100,081; 37,225 fabrication workers and 62,856 non-fabrication workers)	58% of workers were born after 1959		2,644	0.54 (0.52, 0.56)	832	0.73 (0.68, 0.78)	35	0.77 (0.54, 1.07)
		Vital status data: 1983-2007								
		Follow-up: 1,490,486 PYs								
Lee et al., 2011 [14]	Korea	Workers in 8 semiconductor factories in Korea (n=113,443, 1998-2008)	Most common age group as of 2009: 30-39 yr for men, 20-29 yr for women	Men workers (n=48,589)	153	0.25 (0.21, 0.29)	48	0.44 (0.32, 0.58)	3	0.39 (0.08, 1.14)
		Follow-up: 832,512 PYs		Women workers (n=64,854)	114	0.66 (0.55, 0.80)	24	0.79 (0.51, 1.18)	7	1.37 (0.55, 2.81)
		Mean length of follow-up: 7.3 yr								

SMR, standardized mortality ratio; CI, confidence interval; PYs, person-years.

1 Adjusted for the deprivation index.

2 Health and Safety Executive reports (2001 and 2010) are from the same study.

3 Sorahan et al. [3] and Sorahan et al. [5] are previous reports of the same study.

**Table 2.** Estimation of the relative SMR for all cancers and leukemia

Study		All causes		All cancers		All causes other than all cancer		rSMR (95% CI)	Leukemia		All causes other than leukemia		rSMR (95% CI)
		Obs	Exp	Obs	Exp	Obs	Exp		Obs	Exp	Obs	Exp	
McElvenny et al., 2003 [6]^1^	Men	27	68	6	13	21	55	1.24 (0.41, 3.14)	-	-	27	68	-
	Women	44	59	23	21	21	38	2.00 (1.06, 3.79)	1	1	43	58	2.34 (0.06, 13.73)
Beall et al., 2005 [7]	Worker^2^	6,579	10,122	2,159	2,765	4,420	7,354	1.30 (1.23, 1.37)	91	107	6,488	10,015	1.31 (1.05, 1.61)
Nichols et al., 2005 [9]^3^	All	307	385	111	145	196	241	0.95 (0.74, 1.20)	3	3	304	382	1.21 (0.25, 3.56)
Hsieh et al., 2005 [8]	Men	98	363	27	66	71	297	1.72 (1.06, 2.71)	7	3	91	360	8.62 (3.37, 18.49)
	Women	93	148	23	34	70	114	1.11 (0.66, 1.80)	-	-	-	-	-
Boice et al., 2010 [11]	All	2,664	4,960	832	1,142	1,832	3,820	1.52 (1.40, 1.65)	35	46	2,629	4,915	1.44 (1.00, 2.01)
Lee et al., 2011 [14]	Men	153	612	48	109	105	503	2.11 (1.47, 2.99)	3	8	150	604	1.57 (0.32, 4.68)
	Women	114	173	24	31	90	142	1.22 (0.74, 1.93)	7	5	107	168	2.15 (0.84, 4.58)

SMR, standardized mortality ratio; rSMR, relative standardized mortality ratio; CI, confidence interval; Obs, observed; Exp, expected.

1 Health and Safety Executive reports (2001 and 2010) are from the same study.

2 Workers at semiconductor facilities at East Fishkill, Burlington, and San Jose.

3 Sorahan et al. [3] and Sorahan et al. [5] are previous reports of the same study.
